# Modeling household adoption of IoT-based home security in Dhaka: a PLS–machine learning framework

**DOI:** 10.3389/fdata.2026.1718710

**Published:** 2026-02-04

**Authors:** Arif Mahmud, Ashikur Rahman, Fahmid Al Farid, Jia Uddin, Hezerul Bin Abdul Karim

**Affiliations:** 1 Department of Computer Science and Engineering, Daffodil International University, Dhaka, Bangladesh; 2 Department of Information Technology & Management, Daffodil International University, Dhaka, Bangladesh; 3 Centre for Image and Vision Computing (CIVC), COE for Artificial Intelligence, Faculty of Artificial Intelligence and Engineering (FAIE), Multimedia University, Cyberjaya, Malaysia; 4 AI and Big Data Department, Endicott College, Woosong University, Daejeon, Republic of Korea

**Keywords:** attitude-social influence-self-efficacy, home security, internet of things, PLS-ML, protection motivation theory

## Abstract

**Introduction:**

Despite several strategies, Bangladesh has a poor rate of internet of things (IoT) deployment. This study therefore seeks to investigate the factors shaping IoT adoption for residential security in Dhaka and to analyze their respective contributions.

**Method:**

Hence, this study combined two important theories, namely protection motivation theory (PMT) along with attitude-social influence-self-efficacy (ASE) in which a hybrid PLS-Machine learning approach has been used to identify both linear and nonlinear correlations with high predictive accuracy. Snowball sampling method was utilized to choose 348 valid replies from a survey of household heads. Afterward, partial least squares (PLS) followed by artificial neural networks (ANN) and machine learning (ML) classifiers were the procedures that made up the complete assessment method.

**Results:**

The variables that affected intention with a variance of 34.9% and accuracy of 74.28% were severity, vulnerability, response efficacy, response cost, and attitude. On the other hand, vulnerability was the most significant predictor, followed by response cost, attitude, response efficacy, self-efficacy, social influence, and severity.

**Discussion:**

The theoretical contribution of this study lies in its novel integration of PMT and ASE models, offering new insights into their combined effect on technology adoption in emerging markets. Besides, the findings contribute to the literature by increasing the public awareness of home security that can enhance Dhaka's overall state of public order and safety. Moreover, the findings may offer valuable insights for companies and entrepreneurs, as incorporating these factors into marketing strategies and investment initiatives is likely to foster greater consumer adoption.

## 1 Introduction

The Internet of Things (IoT) has profoundly impacted the evolution of Information and Communication Technology. The emergence of compact, internet-connected, and wireless sensors has not only transformed how data is ubiquitously collected but has also instilled a vision of “smartness” across various environments (Ahanger et al., 2020). With the global IoT market projected to exceed USD 1.6 trillion by 2025 and over 34 billion connected devices worldwide (Anddresey et al., 2025), innovations in smart home technologies are transforming residential security; in Dhaka, understanding the factors influencing the adoption of IoT-enabled home security solutions is essential for enhancing household safety and supporting broader public security efforts.

Home automation systems involve the automated control and programmable operation of various household appliances and devices, thereby minimizing the need for manual interaction in managing daily domestic tasks (Sayeduzzaman et al., 2024). These interconnected sensors and devices leverage an IoT-enabled platform, granting users global connectivity and command. This pervasive interconnectivity allows smart home devices to gather real-time data from diverse sources, significantly enhancing both user safety and overall security (Sobhan et al., 2025). One of the most critical applications of these systems is safety and security, especially given the recent surge in burglaries, thefts, and other security incidents that threaten personal wellbeing (Siwakoti et al., 2023).

Beyond automation and security, smart home technology also facilitates efficient energy management, offering an effective method for optimizing energy use within residential structures (Ejaz et al., 2017). With rapid advancements in smart home technology coinciding with growing populations, these systems are becoming crucial for optimizing residential electricity consumption (Netinant et al., 2024). Currently, over 100 million households utilize IoT security devices, a figure expected to triple by the decade's end (Anddresey et al., 2025). In developed countries, IoT adoption at the household level has become increasingly common, with smart appliances, security systems, and energy management solutions integrated into everyday life (Sámano-Ortega et al., 2023). By contrast, in many developing countries, household use of IoT remains constrained by affordability, lack of infrastructure, digital literacy gaps, and uneven internet access (Hossain et al., 2024).

Bangladesh is experiencing significant growth in digital connectivity, providing a foundation for the adoption of IoT applications including home security. As of 2025, internet access in Bangladesh has expanded to 54.8% of households, showing steady growth from 38.1% in 2022 (Rahman et al., 2025). The smart home market in Bangladesh is expected to grow from US$476.6 million in 2024 to around US$536 million in 2025, continuing at a 12.4% annual growth rate and reaching US$760.5 million by 2028 (Hossain et al., 2024). This growth in connectivity and device availability indicates a stronger basis for the adoption of IoT-based solutions in the country. Although IoT adoption in Bangladesh is still at an early stage, it demonstrates considerable potential for transforming key sectors such as smart cities, healthcare, traffic management, and home automation (Hossain et al., 2025). Government initiatives and research projects are laying the groundwork for wider integration, signaling strong interest in leveraging IoT for socio-economic development. Despite these efforts, the drivers of adoption remain largely unexamined, creating opportunities for researchers to conduct holistic studies to uncover underlying factors and theoretical foundations.

The Protection Motivation Theory (PMT) is a practical and reliable social theory used to assess fear, danger, and threats (Westcott et al., 2017). This assertion is supported by prior studies (Srisawang et al., 2015; Jansen and van Schaik, 2018), which emphasize that PMT can successfully address crime- and security-related issues. The Attitude-Social Influence-Self-Efficacy (ASE) model also aids in identifying and predicting factors that affect a consumer's acceptance of new technology (Tani et al., 2022; Shabiq and Hassan, 2016; Aziz et al., 2017). The integration of these two models can improve the identification of factors that spur the adoption of IoT-enabled security devices. If a comprehensive IoT-enabled security system is implemented in Dhaka city households, it is projected that burglaries and other criminal attempts against homes would be minimized. To increase IoT adoption for home security, it is also crucial to recognize the influencing elements and each one's unique contribution.

Although previous studies have explored the adoption of IoT technologies in various contexts (Negm, 2023; Rodić et al., 2023; Zhang and Lee, 2023), factors specifically influencing the adoption of IoT-enabled home security systems remain underexamined. While Protection Motivation Theory (PMT) and the Attitude-Social Influence-Self-Efficacy (ASE) model have been widely applied in other domains (Mahmud et al., 2024b; Mou et al., 2022; Saygılı et al., 2022; Mahmud et al., 2022b), their integration within the context of IoT security has not been sufficiently investigated. Additionally, despite the rise of IoT security solutions globally (Zhang and Lee, 2023; Li et al., 2024), empirical studies examining the determinants of adoption in developing countries, like Bangladesh, are limited. While theories like TAM (Technology Acceptance Model) and UTAUT (Unified Theory of Acceptance and Use of Technology) are widely used to explain technology adoption (An et al., 2023; Menon and Shilpa, 2023), they tend to focus more on the perceived ease of use, usefulness, and social influences without adequately addressing how fear of security threats or individual coping capabilities influence technology acceptance. Besides, Technology Threat Avoidance Theory (TTAT) is new, inconsistent and needs to be widely explored (Carpenter et al., 2019). Moreover, this model places excessive emphasis on individuals' perceptions and thoughts, rather than addressing the technical and organizational viewpoints. According to Westcott et al. (2017) and Haag et al. (2021), PMT is allied to threat-related research where appropriate solutions are available to individuals. In addition, Ifinedo (2012) points out that attitude and social influence are also important predictors of the intent to adopt a security system. However, importantly, attitude and social influence are the two variables that are ignored in PMT.

This study addresses these gaps by integrating PMT and ASE models to understand the specific factors influencing IoT adoption for home security in Dhaka. By combining these models, this research provides a more comprehensive framework for understanding consumer behavior, offering insights into how perceived severity, vulnerability, response efficacy, response cost and self-efficacy, alongside social influences and attitudes, impact adoption intentions. On the other side, consumer, student, professionals are the majority samples used in Bangladesh context (Hassan et al., 2022; Rahman and Nasrin, 2024; Emon et al., 2024). Bangladeshi family heads are less studied sample types despite being the ultimate decision maker within the family. Last but not least, (Mahmud et al. 2024a), (Al-Skaf et al. 2021), and (Duc et al. 2023) have integrated PLS-SEM with ML, there hasn't been much experimentation with the hybrid PLS-ML model in the context of the IoT for security objectives. This indicates clear gaps in the current literature, underscoring the necessity for focused research in these areas. Given this gap in the literature, the current study aims to address these challenges by applying a hybrid PLS-SEM and machine learning approach to explore the factors affecting IoT adoption for home security. The study's objectives are threefold:

- **RO1:** To determine the factors that influence the adoption of IoT.
- **RO2:** To determine the contribution of each factor in the adoption of IoT.
- **RO3:** To predict the accuracy of the adoption of IoT.

The structure of this paper is organized into multiple sections. After the introduction, Section 2 identifies the literature gap and presents the literature review. Section 3 proposes the conceptual model based on theoretical justifications and hypotheses. Section 4 details the methodology employed. Sections 5, 6 present the study's findings and discussion, respectively. Finally, the research's contributions, limitations, future directions, and conclusions are explored in Section.

## 2 Literature review

**Figure 1:**
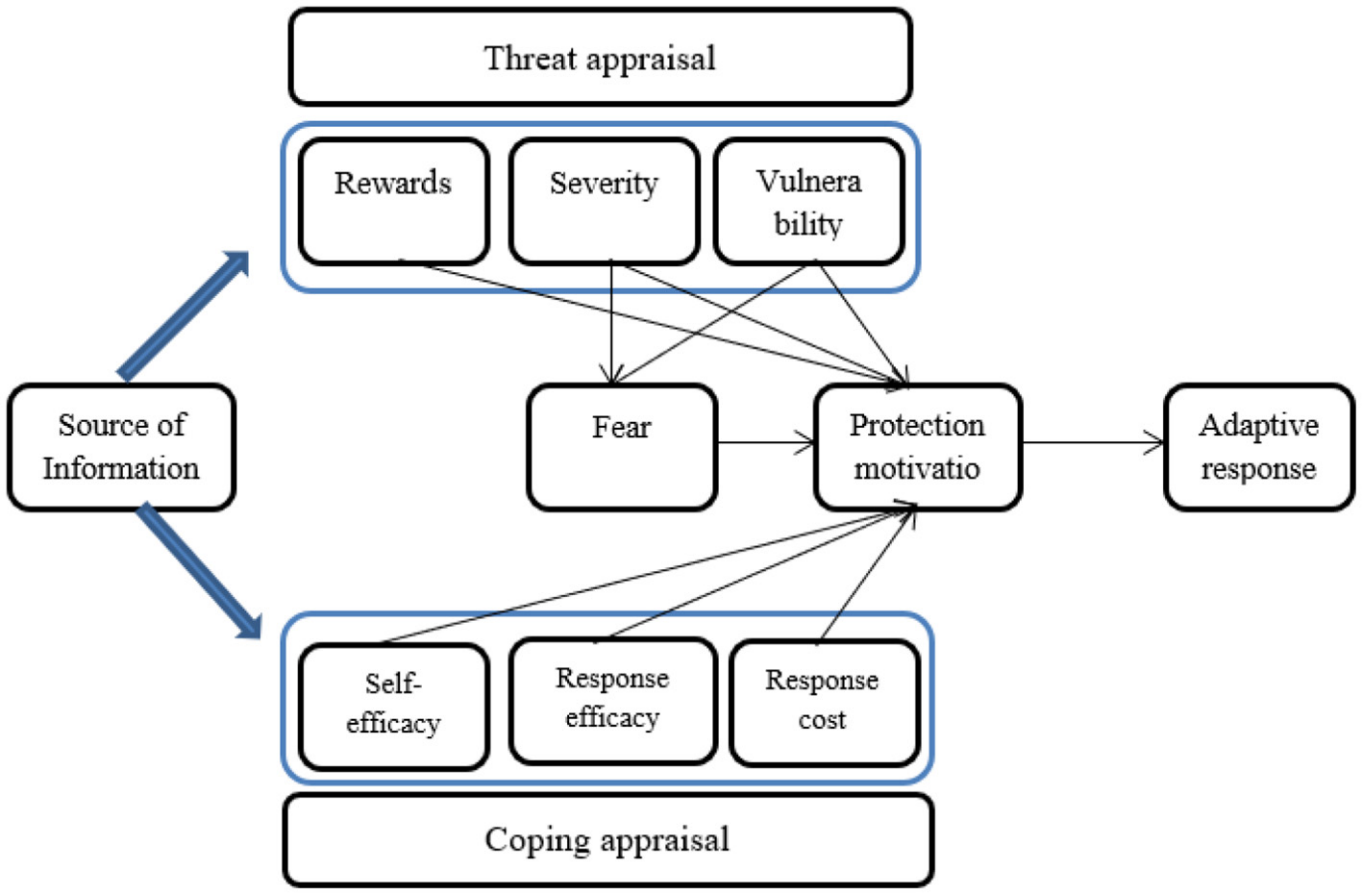
Structure of PMT (Posey et al., 2015).

Based on the concept of the parallel process model, Rogers introduced PMT in 1975 (Verkijika, 2017). According to Figure 1, the outcomes of cognitive processes can manifest as adaptive or maladaptive reactions. Adaptive reactions are techniques that positively lessen the danger. Maladaptive reactions, on the other hand, may diminish fear but fall short of genuinely minimizing the hazard (Posey et al., 2015). Additionally, the sources of knowledge for these processes might be interpersonal, environmental, etc. Vulnerability, severity, and intrinsic or extrinsic benefits of using maladaptive reactions contribute to the development of threat evaluation. Contrarily, coping evaluation for using the adaptive coping strategy takes into account self-efficacy, response cost and response efficacy (Posey et al., 2015; Aurigemma and Mattson, 2018). The intention to carry out the desired conduct that results from these two evaluation processes is known as protection motivation, which is often equivalent to the behavioral intention (Verkoeyen and Nepal, 2019).

While Protection Motivation Theory (PMT) is traditionally applied to health-related contexts (Chiu et al., 2025; Gholian-Aval et al., 2025; Gopal and Thomas Gil, 2025), this study adapts its framework to better understand threat perception in the adoption of technology-mediated solutions, such as IoT devices for home security. In classical health-psychology models, threat appraisal and coping appraisal are central to the decision to adopt protective behaviors (Verkoeyen and Nepal, 2019; Posey et al., 2015). In the context of IoT adoption, threat perception operates differently than in traditional health contexts. In this study, we focus on how threat appraisal—specifically, perceived vulnerability and perceived severity—influences the intention to adopt IoT-based home security systems. IoT devices, such as smart cameras and sensors, are seen as tools that can effectively counteract the perceived threats, providing a sense of control and protection. In the context of IoT adoption for home security, perceived vulnerability refers to the individual's belief about the likelihood of experiencing a digital security threat, such as a data breach, hacking, or device malfunction. Perceived severity, on the other hand, reflects the seriousness of these potential threats—for example, the loss of personal data, privacy violations, or financial loss resulting from unauthorized access. These perceptions drive consumers' motivation to adopt IoT technologies, as they seek to mitigate these digital risks. Thus, PMT applies by emphasizing how security concerns shape the decision to adopt technology. This aligns with PMT's coping appraisal, where individuals evaluate the response efficacy and response cost.

Recent extensions of Protection Motivation Theory (PMT), such as those by Liang and Xue (2010) and Warkentin et al. (2016), emphasize the affective dimension of fear appeals, which focus on emotional responses to digital threats like data breaches or cyberattacks. In the context of IoT adoption, individuals may experience fear, anxiety, or concern about the security risks associated with these technologies, influencing their threat appraisal. This emotional reaction can intensify the perceived severity and vulnerability of potential risks, motivating consumers to adopt security technologies to alleviate these feelings. Therefore, the affective dimension of fear appeals plays a crucial role in shaping consumers' coping behaviors, as emotional reactions often drive individuals to seek out technologies that promise to reduce perceived risks and emotional discomfort.

However, among 138 studies, only 13 and 12, respectively, have treated threat and coping appraisal as distinct variables inside PMT. Severity, self-efficacy, response cost, vulnerability, and response efficacy, on the other hand, have each been used in articles in a manner that ensures that these variables are most frequently and effectively used (Mahmud et al., 2022a). Threat and coping assessments, however, have often been treated as independent processes rather than as separate entities (Giwah et al., 2019). Furthermore, Mahmud et al. (2022a) reveal that threat appraisal is comprised of severity and vulnerability whereas coping appraisal is made up of self-efficacy, response cost and response efficacy. Although Protection Motivation Theory (PMT) research frequently incorporates mediation or moderation mechanisms, this study intentionally focuses on direct structural relationships. The primary aim is predictive modeling of IoT-enabled home security adoption rather than testing complex causal pathways. Incorporating mediation or moderation effects would increase model complexity and risk overfitting, which could compromise predictive stability, particularly when integrating PLS–SEM with machine learning algorithms.

Furthermore, both Boss et al. (2015) and Posey et al. (2015) have disregarded the mediating role of threat and coping assessment. Considering that the study seeks to examine the predictors of intention, only the independent variables of PMT have been inherited and the mediation effect has been ignored. . Consistent with prior prediction-oriented PMT studies, direct effects were therefore emphasized. Here's a list of a few recent papers where the variables of PMT have been used as direct predictors of intention that justifies the claim of study (see Table 1). A significant contribution across these studies is the integration of cultural and psychological factors in shaping security behaviors. (Ameen et al. 2019), (2021) and Crossler et al. (2019) highlight how cultural dimensions like individualism and collectivism impact cybersecurity compliance, offering a nuanced understanding of security behaviors in different cultural settings. Farooq et al. (2019) and Kusyanti et al. (2019) examine security behaviors in developing countries like Kenya and Indonesia, emphasizing the role of socio-cultural influences in shaping security intentions, though their findings contradict typical PMT assumptions. For instance, Farooq et al. (2019) found that factors like perceived vulnerability and severity did not significantly influence security behaviors, contrary to expectations from PMT. These contradictions highlight a gap between theoretical assumptions and real-world behavior. Furthermore, many studies underscore limitations in terms of sample size and geographical focus. For example, studies by (Ameen et al. 2019), (2021) focus on specific regions like the UAE and US, limiting the generalizability of findings. Several papers also point to a gap in understanding how to effectively apply theoretical models in improving security compliance, especially in resource-limited settings like Palestinian universities (Iriqat et al., 2019) and developing countries (Hina et al., 2019).

**Table 1:** Variables of PMT as direct predictors of intention.

**No**	**References**	**Country**	**Sources**	**Application**	**Independent/Dependent variables**
1	Ameen et al., 2019	UK, Oman	Comp. in Human Beh.	Smartphone security	PS, PV, PSE, PRE, RC, PCS, SAC, UA, PD, IVC, MVF / INT
2	Farooq et al., 2019	Finland, Kenya	IEEE AFRICON Conference	Security behavior	PS, PV, PSE, PRE, RC, ATT, SN, SS, DN / INT, BHV
3	Wang, 2020	Taiwan	Int. J. of Bank Marketing	M-payment security	PS, PV, PSE, PRE, RC, PB / INT
4	Al-Emran et al., 2020	UK, Oman, USA	Comp. in Human Beh.	Cyber-security compliance	PS, PV, PSE, PRE, RC, ATT, SN, CYPO, TMGT, PTH, SANC / INT, BHV
5	Yang et al., 2020	US	AIS transactions on replication research	InfoSec protection	PS, PSUS, PSE, PRE, RC, PAUTO, PREL, PCOM, RPM / INT
6	Crossler et al., 2019	US	Information & management	Protective info technologies	PS, PV, PSE, PRE, RC / INT
7	Hina et al., 2019	Malaysia, US	Computers & security	Security compliance behavior	PS, PV, PSE, PRE, SN, ATT / INT
8	Hooper and Blunt, 2019	New Zealand	Behavior & info technology	InfoSec behavior	PSE, PRE, RC, SN, PB, LIKE, IMP, DET, SANC / INT
9	Iriqat et al., 2019	Malaysia	ICOICE 2019 Conference	InfoSec policy	SANCC, PS, PSE, RE, PRE, INFQ, PRI, FC / INT
10	Kusyanti et al., 2019	Indonesia	Procedia computer science	Protecting Facebook password	PS, PV, PSE, PRE, RC, PSUS, FEAR, PEX, SN, PRES, PSS / INT
11	Rajab and Eydgahi, 2019	US	Computers & security	InfoSec policies	PS, PV, PSE, PRE, RC, ATT, PBC, SN, AWARE, SANC, DC, SC, TMS, PEPR, ORGC / INT
12	Al-Emran et al., 2020	Vietnam, Croatia, Malaysia, UK, Oman	J. of enterprise info management	Smart-watch security	PS, PV, PSE, PRE, RC, PU, PEOU / INT
13	Mahmud et al., 2023	Bangladesh, Malaysia	J. of systems and info technology	IoT security devices	PS, PV, PSE, PRE, RC / INT
14	Marikyan et al., 2022	UK	Computers in human behavior	Blockchain adoption	PS, PV, PSE, PRE, RC/INT
15	Kimpe et al., 2021	Belgium, UK	Behavior & info technology	Cyber-security	PS, PV, PSE, PRE / INT
16	Skalkos et al., 2021	Greece	J. of cybersecurity and privacy	Biometrics authentication	PS, PV, PSE, PRE, RC, INNOV/INT

Variables: INT, Intention; BHV, Behavior; PSE, Perceived self-efficacy; PS, Perceived severity; PRE, Perceived response efficacy; PV, Perceived vulnerability; RC, Response cost; UA, Uncertainty avoidance; PD, Power distance; IVC, Individualism vs collectivism; MVF, Masculinity vs. femininity; PCS, Perceived certainty of sanction; SAC, Severity of adverse consequences; ATT, Attitude; SN, Subjective norm; SS, Social support; DN, Descriptive norms; PB, Perceived benefit; CYPO, National smartphone cybersecurity policies; TMGT, Top-management participation; PTH, Perceived threat; SANC, Sanctions; PSUS, Perceived threat susceptibility; PAUTO, Perceived autonomy; PREL, Perceived relatedness; PCOM, Perceived competence; RPM, Response performance motivation; LIKE, Likelihood; IMP, Impact; DET, Detection; SANCC, Sanction certainty; INFQ, Info-Quality; PRI, Perceived privacy; FC, Facilitating condition; FEAR, Fear; PEX, Prior experience; PSS, Perceived Security (Support); PRES, Personal responsibility; PBC, Perceived behavior control; AWARE, Awareness; SC, Sanction celerity; TMS, Top Management Support; PEPR, Peer Pressure; ORGC, Organizational Climate; DC, Perceived certainty of detection; PU, Perceived usefulness; PEOU, Perceived ease of use; INNOV, Innovativeness.

Mahmud et al. (2023) and Marikyan et al. (2022) apply PMT to emerging technologies like IoT and blockchain. Mahmud et al. emphasize perceived vulnerability and self-efficacy in Generation Z's adoption of IoT security measures, highlighting gender differences, while Marikyan et al. show that self-efficacy and response efficacy predict blockchain adoption. In contrast, Rajab and Eydgahi (2019) focus on PMT's applicability in higher education, finding that PMT best explains compliance intentions, though they find limited support for other frameworks like General Deterrence Theory (GDT). Skalkos et al. (2021) explore Behavioral Biometrics Continuous Authentication (BBCA), showing that privacy concerns and trust significantly influence adoption intentions, but leaving a gap in understanding trust dynamics. Wang (2020) on mobile payments finds that self-efficacy and response efficacy are crucial in adoption, but risk appraisal does not have a significant impact, which contrasts with Mahmud et al.'s emphasis on perceived vulnerability. Yang et al. (2020) compares organizational and home users, revealing that organizational users are more motivated by self-determined appeals (SDT) rather than fear-based PMT appeals.

A significant contribution across these studies is the integration of cultural and psychological factors in shaping security behaviors. Crossler et al. (2019) highlight how cultural dimensions like individualism and collectivism impact cybersecurity compliance, offering a nuanced understanding of security behaviors in different cultural settings. Farooq et al. (2019) examine security behaviors in developing countries like Kenya and Indonesia, emphasizing the role of socio-cultural influences in shaping security intentions, though their findings contradict typical PMT assumptions. For instance, Farooq et al. (2019) found that factors like perceived vulnerability and severity did not significantly influence security behaviors, contrary to expectations from PMT. These contradictions highlight a gap between theoretical assumptions and real-world behavior. Furthermore, many studies underscore limitations in terms of sample size and geographical focus. For example, studies by Ameen et al. (2019) focus on specific regions like the UAE and US, limiting the generalizability of findings. Several papers also point to a gap in understanding how to effectively apply theoretical models in improving security compliance, especially in resource-limited settings like Palestinian universities (Iriqat et al., 2019) and developing countries (Hina et al., 2019).

Mahmud et al. (2022a) and Dupuis and Ebenezer (2018) apply PMT to emerging technologies like IoT and blockchain. Mahmud et al. (2024b) emphasize perceived vulnerability and self-efficacy in Generation Z's adoption of IoT security measures, highlighting gender differences, while Dupuis and Ebenezer (2018) show that self-efficacy and response efficacy predict blockchain adoption. In contrast, Ejaz et al. (2017) focus on PMT's applicability in higher education, finding that PMT best explains compliance intentions, though they find limited support for other frameworks like General Deterrence Theory (GDT). Skalkos et al. (2021) explore Behavioral Biometrics Continuous Authentication (BBCA), showing that privacy concerns and trust significantly influence adoption intentions, but leaving a gap in understanding trust dynamics. Wang (2020) on mobile payments finds that self-efficacy and response efficacy are crucial in adoption, but risk appraisal does not have a significant impact, which contrasts with Mahmud et al. (2024b)'s emphasis on perceived vulnerability. Yang et al. (2020) compares organizational and home users, revealing that organizational users are more motivated by self-determined appeals (SDT) rather than fear-based PMT appeals.

**Figure 2:**
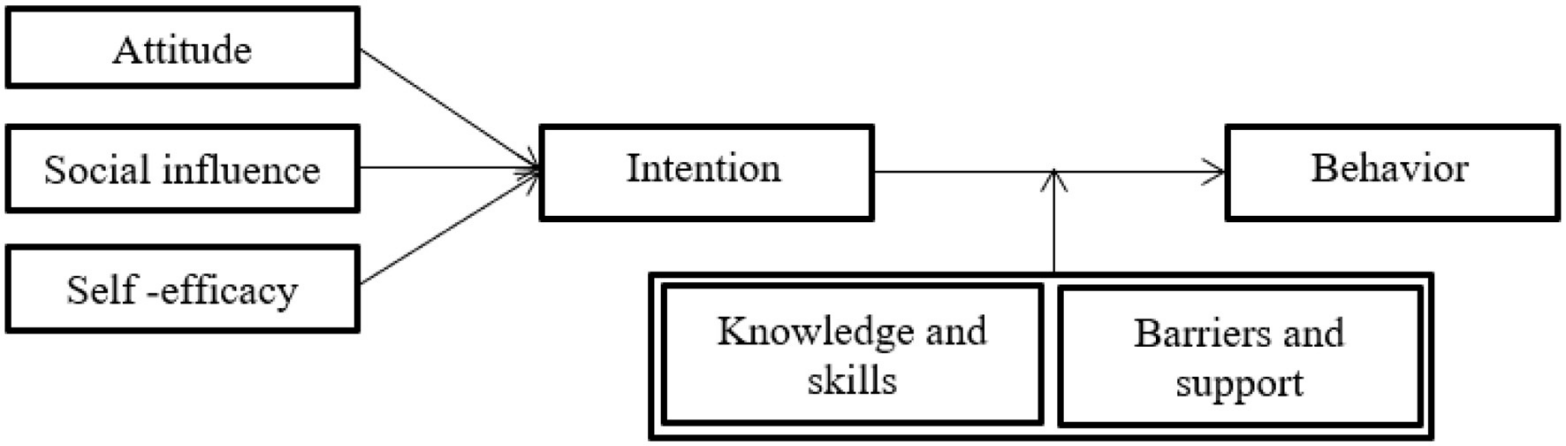
Structure of ASE (Zwerver et al., 2011).

Ajzen (1991) said that people are more likely to sustain the behavior when their attitude and social influence increase. Self-efficacy also relates to beliefs about one's capacity to carry out particular activities under particular circumstances (de Vries et al., 1988). Based on these ideas, in 1988, de Vries created the ASE model, which combines the concepts of the two widely used models, the TRA and social learning theory. According to the model (see Figure 2), an individual's decision to engage in an activity is characterized by attitude, social influence, and self-efficacy (Merkx et al., 2017).

By combining PMT and ASE, this study provides a more holistic model for understanding IoT adoption. While PMT focuses on fear-driven motivation based on perceived threats (e.g., vulnerability to burglary), the ASE model complements this by showing how self-efficacy and attitudes toward technology mediate the adoption process. This dual framework enriches theoretical discussions by explaining not only the emotional triggers that drive adoption (e.g., fear of security threats) but also the cognitive factors (e.g., perceived ability and attitudes) that shape how individuals respond to those threats. The integration of these two models allows for a more nuanced understanding of how motivation and fear interact, which is crucial for explaining the decision to adopt IoT technology in security applications. Moreover, this combination allows for a more holistic view of the complex factors that influence consumers' decisions to adopt IoT technologies, particularly in the context of home security in Dhaka

## 3 Hypotheses and model development

Burglaries can instill a significant fear of property loss within households (Mahmud et al., 2023). Kim and Kyung (2023) suggest that the likelihood of adopting IoT devices increases with the severity of the perceived threat. Furthermore, greater perceived threat severity associated with an IoT device has been shown to increase the likelihood of engaging in behaviors aimed at mitigating this risk, as evidenced by Dupuis and Ebenezer (2018). Additionally, this factor affects the intention to adopt mobile banking apps and smart-watch favorably (Kala et al., 2021; Al-Emran et al., 2020). Similarly, the following hypothesis can be proposed:

**H1:** The IoT adoption intention for home security is positively influenced by perceived severity.

According to Al-Emran et al. (2020), people might be more motivated to prevent security breaches if they had a high impression of security vulnerabilities. According to Wang (2020), users who believe that recommended security measures effectively mitigate threats are more likely to adopt and comply with these measures. Likewise, the prevalence of home burglaries may drive residents in Dhaka to adopt IoT security solutions (Mahmud et al., 2023). Moreover, followed by Kala et al. (2021), this variable positively impacts the individuals' behavioral intentions to adopt mobile apps and m-payment (Wang, 2020). On the basis of the foregoing discussion, the following hypothesis is advanced:

**H2:** The IoT adoption intention for home security is positively influenced by perceived vulnerability.

Security measures leveraging IoT technology are projected to effectively reduce the risk of burglaries (Mahmud et al., 2023). According to Wang (2020), users who believe that recommended security measures effectively mitigate threats are more likely to adopt and comply with these measures. The adoption intention of smart watches (Al-Emran et al., 2020) and m-payments (Wang, 2020) was also found to be positively influenced by this variable. Therefore, we can expect the following:

**H3:** The IoT adoption intention for home security is positively influenced by perceived response efficacy.

Elevated response costs discourage people from engaging in threat-mitigating behaviors (Mahmud et al., 2023). Fei et al. (2022) argue that lower perceived response costs associated with technology adoption lead to increased efforts to engage in behaviors aimed at mitigating the identified threat. Moreover, Al-Emran et al. (2020) and Marikyan et al. (2022) identified that this variable hurt the adoption of smart-watches and blockchain, respectively. Consequently, the following hypothesis can be posited:

**H4:** The IoT adoption intention for home security is negatively influenced by perceived response cost. According to AlHamad et al. (2021), stronger sense of self-efficacy is linked to a heightened motivation to take steps to address and reduce potential threats. Wang (2020) further affirms that this variable plays a pivotal role in information security studies, indicating that a higher level of self-efficacy correlates with greater adoption of technology. Besides, this variable plays an important role in adopting mobile warning systems (Fischer-Preßler et al., 2022) and blockchain (Marikyan et al., 2022). Accordingly, the preceding discussion suggests the following projection:

**H5:** The IoT adoption intention for home security is positively influenced by perceived self-efficacy.

Before using any technology, people frequently consult their friends and relatives who are well-wishers and well-informed about the technologies (Mahmud et al., 2022a). Conversely, this variable has been identified as a significant predictor in information security studies, with higher social influence leading to greater technology adoption (Giua et al., 2022). Furthermore, social influence is observed to exert a positive impact on knowledge-sharing intentions (Kalra and Baral, 2020) and e-trading adoption (Chaudhary and Suri, 2022). So, the following hypothesis can be projected in light of the debate above:

**H6:** The IoT adoption intention for home security is positively influenced by social influence.

Bhalla (2023) highlighted that when customers feel more content, their attitudes would be positively impacted and they would be more inclined to use this service. Similarly, Na et al. (2023) find that when consumers prioritize their well-being, they tend to exhibit a positive attitude toward the service and are more inclined to adopt such services. Additionally, this characteristic is crucial for the uptake of electric vehicles (Khurana et al., 2020) and online shopping (Usman and Kumar, 2021). Hence, the following hypothesis can be suggested:

**H7:** The IoT adoption intention for home security is positively influenced by attitude.

**Figure 3:**
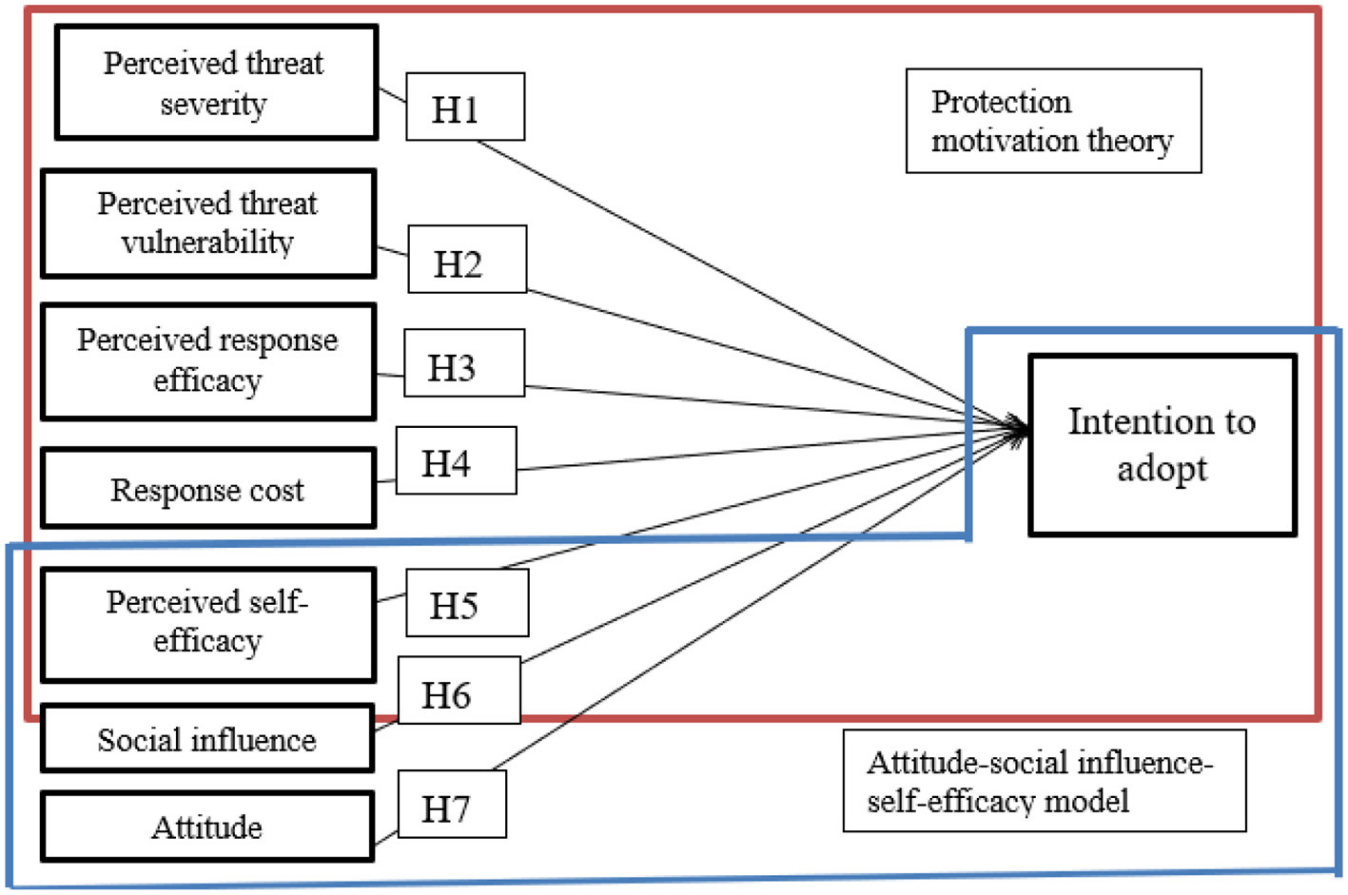
Proposed model.

In order to increase IoT adoption for home security, this study has incorporated the factors of two key theories; PMT and ASE (see Figure 3). Perceived severity, perceived response efficacy, perceived vulnerability, perceived self-efficacy, and response cost are the components of PMT theory. The ASE model, in contrast, includes attitude, self-efficacy and social influence. All these seven variables are directly connected to the intention to adopt IoT which is represented by seven hypotheses (H1-H7).

## 4 Methodology

The household head of Dhaka city who are potential users of IoT for home security are considered as the sample of this research. The results should therefore be interpreted as reflective primarily of urban, digitally aware household heads, which are more likely to engage with and adopt IoT-enabled home security technologies. On the other hand, according to Twine et al. (2019), the age of the household head should be more than 24. It is worth mentioning that even though there are several household heads, data are collected from a single member of a family. In this study, snowball sampling was utilized to collect data. This strategy is especially useful when the target demographic is difficult to reach (Woodley and Lockard, 2016). The respondents in this research are difficult to reach for two reasons. Firstly, respondents have to fulfill several selection criteria and secondly, data are collected during the pandemic times. Data was gathered for about 5 months between 25 March 2021 and 21 August 2021 utilizing both paper questionnaires and online surveys (Google Forms). Most participants favored online questionnaires due to the pandemic scenario in Dhaka during the data collection time.

The snowball process was initially initiated by the researchers who reached out to a small group of known household heads within local communities. These initial participants were then asked to refer others in their social networks who met the study's inclusion criteria. A total of thirty two seed participants were initially recruited, each of whom was asked to refer other household heads. This allowed the snowball process to grow gradually, expanding the sample size. Participants were recruited through local community networks, neighborhood contacts, and word-of-mouth. In addition, most referrals were made via Messenger, Telegram, Email, and WhatsApp. As per inclusion criteria, participants had to be household heads of Dhaka city that were at least 24 years old and had some knowledge or interest in IoT or home security technologies.

The constructs were measured using items derived from previous research. To be more precise, the items of severity, response efficacy, vulnerability, self-efficacy, and response cost were provided by Thompson et al. (2017). Additionally, (Iranmanesh et al. 2017), (Magotra et al. 2016), and (Zhou et al. 2020) were followed to gain items of attitude, social influence, and intention, respectively. All of these constructs including response cost are reflective by nature as adapted from existing research papers. For this cross-sectional study, responses were recorded using Likert scale-7. A cross-sectional design was chosen because the study had a limited timeframe and longitudinal follow-up of the same households was not feasible. It also enabled efficient collection of a sufficiently large sample for robust PLS-SEM estimation and subsequent predictive modeling (do Valle and Assaker, 2016). Cross-sectional surveys provide faster, lower-cost evidence than longitudinal designs (Zangirolami-Raimundo et al., 2018). On the basis of Mohammed et al. (2016) suggestions, a pre-test (8 people) and pilot surveys (35 people) were carried out to validate survey items prior to the final survey. The sample size of 35 for pilot test followed Mooney and Duval (1993) who recommended limiting the response between 30 and 50. In the pre-test phase, volunteers verified both the language and length of the measurement items. However, the pilot test results led to the removal of items PS6 and PSE1. The decision to remove these items was primarily based on semantic issues in which these items unclear or difficult to interpret, which may have led to inconsistent responses. We also ensured that the remaining items showed acceptable levels of internal consistency, which was verified through reliability checks in the final survey. The study's minimum sample size, previously computed with G*Power 3.1, was 103. In G*Power, we specified the parameters based on the following: a moderate effect size (*f*^2^ = 0.15), statistical power of 0.80, and an alpha level of 0.05 as followed by Memon et al. (2020). The survey links and printed questionnaire were distributed to 700 persons approximately, who returned 531 copies. Afterward, 348 replies were chosen for the final analysis from the obtained data.

As per Table 2, there were 248 men and 100 women out of 348 participants. In addition, more than 96% of the total respondents were married. On the other hand, around 70% of participants were aged between 36 and 55. Further, more than 50% of the participants attained a bachelor's degree.

**Table 2:** Demographic data.

**Variables**	**Category**	**Frequency**	**Percentage**
Gender	Women	100	28.7
	Men	248	71.3
Age	25–35 years	82	23.6
	36–45 years	170	48.9
	46–55 years	71	20.4
	56–65 years	22	6.3
	66 years and above	3	0.9
Marital position	Married	335	96.3
	Single	13	3.7
Academic certificate	Post-Doctoral certificate	2	0.6
	Ph.D. certificate	8	2.3
	Masters certificate	84	24.1
	Bachelor certificate	94	27.0
	Diploma certificate	64	18.4
	Higher secondary certificate	74	21.3
	Secondary school certificate	16	4.6
	No academic certificate	6	1.7

This study used both SPSS and SmartPLS for data analysis. SPSS is well-suited for performing basic statistical analysis such as descriptive statistics, correlation, and reliability testing, which are essential for the initial stages of data examination and scale validation (Rahman and Muktadir, 2021). While SPSS alone does not support more advanced techniques like PLS-SEM, it was utilized for initial steps, and PLS-SEM analysis was conducted separately using SmartPLS due to its ability to model complex relationships and latent variables efficiently (Hair and Alamer, 2022). On the other side, PLS-SEM is preferred over CB-SEM because selected because it is prediction-oriented, suitable for complex models and required less sample sizes (Dash and Paul, 2021). This combination of SPSS for preliminary analysis and SmartPLS for the structural equation modeling is methodologically sound and allows for comprehensive data analysis.

## 5 Results

Data preparation, PLS, ANN, and ML classifiers were the four processes that made up the entire assessment procedure.

### 5.1 Data preparation

SPSS was used to assess these 4 methods, common method variance using Harman's single-factor test, missing data using expectation-maximization, outliers using 5% trimmed means and normality using descriptive statistics. First, Harman's single-factor test yielding approximately 15.56%, indicating that common method variance was not a concern. On the other hand, the majority of respondents preferred online surveys where it was required to complete the entire Google form. There were thus no issues with missing data. To prevent outlier influence, the study adhered to Ahanger et al. (2020) method of keeping a modest gap between the original and 5% trimmed means. Finally, the result of descriptive statistics confirms that the data distribution is non-normal, where large portion of the data presented skewness and kurtosis above the recommended threshold, –3 to +3. Therefore, it shows that the data normality distribution assumption was violated; thus, further supporting the use of PLS-SEM PLS-SEM (Hashim et al., 2023).

### 5.2 Partial least squares

Due to its popularity among academics, the SmartPLS v3.3.3 program was employed to assess our suggested conceptual model using PLS (Wong, 2013). To improve AVE values, 4 items (PS1, PRE1, PSE3, and PS4) were eliminated and latent variable remain intact with rest other variables. Table 3 demonstrates that every requirement was satisfied. Notably, RC1 (0.495) was not deleted for 2 reasons; first it is too close to 0.5 and scores greater than 0.4 are considered stable and can be accepted (Samuels, 2017). To improve AVE values, 4 items (PS1, PRE1, PSE3, and PS4) were eliminated. Table 3 demonstrates that every requirement was satisfied (Samuels, 2017).

**Table 3:** Measurement model assessment.

**Constructs**	**Items**	**Factor loadings**	**VIF**	**VIF (construct)**	**CR**	**AVE**
Adoption intention (AI)	AI1	0.739	1.383	N/A	0.829	0.549
	AI2	0.682	1.391			
	AI3	0.751	1.395			
	AI4	0.788	1.542			
Attitude (ATT)	ATT1	0.876	2.618	1.216	0.934	0.779
	ATT2	0.875	2.684			
	ATT3	0.896	2.490			
	ATT4	0.885	2.887			
Perceived response efficacy (PRE)	PRE2	0.711	1.196	1.448	0.788	0.553
	PRE3	0.738	1.163			
	PRE4	0.781	1.275			
Perceived severity (PS)	PS2	0.725	1.099	1.067	0.760	0.513
	PS3	0.716	1.177			
	PS5	0.707	1.145			
Perceived self-efficacy (PSE)	PSE2	0.905	2.007	1.450	0.916	0.733
	PSE4	0.820	2.637			
	PSE5	0.793	2.950			
	PSE6	0.902	3.318			
Perceived vulnerability (PV)	PV1	0.735	1.520	1.431	0.862	0.510
	PV2	0.696	1.534			
	PV3	0.720	1.594			
	PV4	0.733	1.568			
	PV5	0.725	1.524			
	PV6	0.675	1.385			
Response cost (RC)	RC1	0.495	1.269	1.101	0.903	0.615
	RC2	0.861	2.772			
	RC3	0.894	3.407			
	RC4	0.884	3.205			
	RC5	0.785	2.267			
	RC6	0.713	1.867			
Social influence (SI)	SI1	0.712	2.267	1.404	0.936	0.709
	SI2	0.913	2.721			
	SI3	0.839	3.383			
	SI4	0.821	3.099			
	SI5	0.857	2.540			
	SI6	0.895	3.831			

Each variable's AVE exceeded the highest squared correlation with other variables, confirming adequate discriminant validity (Table 4).

**Table 4:** Discriminant validity.

**Variable**	**AI**	**ATT**	**PRE**	**PS**	**PSE**	**PV**	**RC**	**SI**
AI	0.741							
ATT	0.197	0.883						
PRE	0.443	0.215	0.744					
PS	0.211	0.011	0.176	0.716				
PSE	–0.039	0.308	–0.045	–0.015	0.856			
PV	0.497	0.135	0.520	0.149	–0.021	0.714		
RC	–0.269	0.043	–0.115	–0.192	–0.083	–0.215	0.784	
SI	0.081	0.293	0.045	–0.006	0.513	0.076	–0.049	0.842

The Table 5 shows model fit statistics for AI as the dependent variable. R^2^ (0.349) indicates moderate variance explained, with Q^2^ (0.179) and Q2 Predict (0.318) suggesting moderate predictive relevance. SRMR (0.058) indicates good model fit, and NFI (0.793) shows a reasonable model fit.

**Table 5:** Variance and model fit metrics.

**Dependent variable**	**R^2^**	**R^2^ adjusted**	**Q^2^**	**Q^2^ predict**	**SRMR**	**NFI**
AI	0.349	0.335	0.179	0.318	0.058	0.793

Table 6 shows that five of the seven hypotheses were significant. Severity, response efficacy, vulnerability, attitude and response cost were the factors that influenced intention. On the other hand, self-efficacy and social influence did not influence intention. Thus, H1, H2, H3, H4, and H7 were significant, whereas H5 and H6 were not.

**Table 6:** Hypotheses results.

**No**	**Relationships**	**Path coefficients (β)**	**Mean**	**Standard deviation**	**T values**	***P*-values**	**Remarks**
H1	PS → AI	0.092	0.096	0.046	1.998	0.046	S
H2	PV → AI	0.314	0.313	0.062	5.042	0.000	S
H3	PRE → AI	0.208	0.212	0.065	3.188	0.002	S
H4	RC → AI	–0.171	–0.174	0.049	3.476	0.001	S
H5	PSE → AI	–0.105	–0.070	0.079	1.325	0.186	NS
H6	SI → AI	0.055	0.026	0.084	0.654	0.513	NS
H7	ATT → AI	0.133	0.130	0.046	2.908	0.004	S

S, Significant; NS, Non Significant.

**Figure 4:**
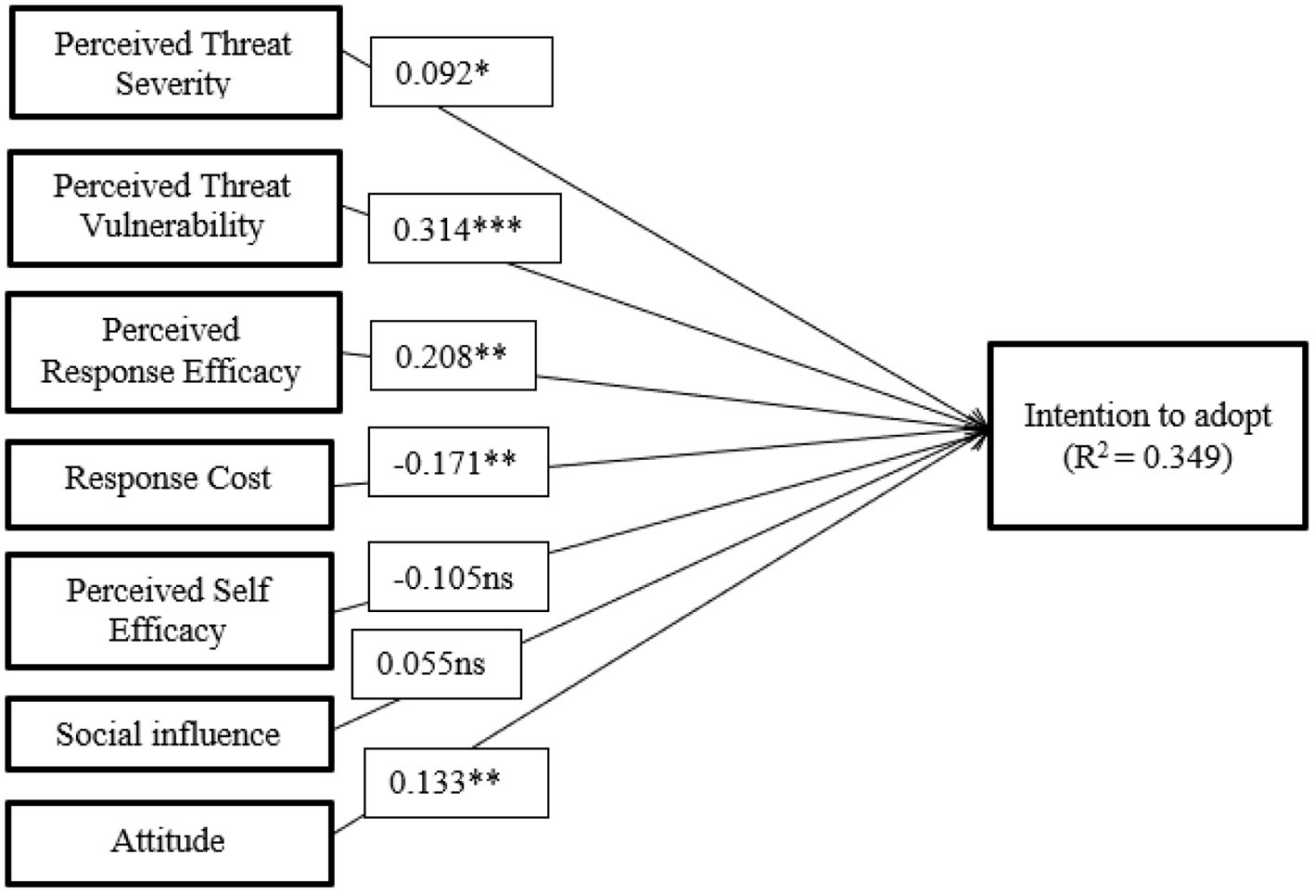
Hypotheses testing results. ns, non-significant; ^*^p < 0.05; ^**^p < 0.01; ^***^p < 0.001.

Effects are categorized as major (≥0.35), medium (≥0.15), small (≥0.02), or very small (≥0.01) (Sawilowsky, 2009). Among the relationships, 4 of them had small effects and 2 of them had very small effects (see Table 7). Figure 4 presents a summary of the hypothesis analysis outcomes. Factors such as attitude (ATT), perceived vulnerability (PV), perceived response efficacy (PRE), and response cost (RC) have a small effect, meaning they slightly influence the likelihood of adopting IoT. Perceived severity (PS) and perceived self-efficacy (PSE) have a very small impact, indicating minimal influence on the decision to adopt. Social influence (SI), however, shows no effect, suggesting that the opinions and behaviors of others do not significantly affect an individual's decision to embrace IoT. Overall, personal attitudes and perceptions about IoT's effectiveness and costs seem to play a more substantial role in adoption than external social pressure.

**Table 7:** Effect size.

**Relationships**	***f*^2^ value**	**Effect**
PS → AI	0.012	Very small
PV → AI	0.106	Small
PRE → AI	0.046	Small
RC → AI	0.041	Small
PSE → AI	0.012	Very small

ns, non-significant, ^*^p < 0.05, ^**^p < 0.01, ^***^p < 0.001.

### 5.3 Artificial neural networks

When the correlations between the variables are linear, partial least squares (PLS) of structural equation modeling is possible to test the hypothesis and performs remarkably well. To avoid producing any unreliable results, it does not take into account the non-linear effects (Zabukovšek et al., 2019). In contrast, ANN can recognize complex non-linear interactions (Leong et al., 2015). Additionally, ANN models can offer greater prediction accuracy than linear models which are extremely durable and versatile (Sim et al., 2014; Tan et al., 2014). However, ANN is not an appropriate method for evaluating the hypotheses (Sim et al., 2014). The employment of PLS and ANN techniques might thus be complementary to one another, according to Zabukovšek et al. (2019). In order to address this limitation, the combined PLS-ANN approach can be used.

Using multilayer perceptrons with sigmoid activation functions (Sharma and Sharma, 2019), the model was trained on 90% of samples and tested on 10% (Leong et al., 2015). Ten-fold cross-validation yielded low RMSE values—0.538 for training and 0.519 for testing—indicating high predictive accuracy and strong model fit (Table 8).

**Table 8:** Validation results.

**Size**	**SSE**	**RMSE**	**Size of samples**	**SSE**	**RMSE**	**Total size**
309	109.377	0.595	39	11.918	0.553	348
314	80.797	0.507	34	11.302	0.577	348
315	84.101	0.517	33	6.558	0.446	348
309	77.272	0.500	39	10.326	0.515	348
305	84.379	0.526	43	11.201	0.510	348
316	105.708	0.578	32	10.249	0.566	348
314	91.157	0.539	34	11.522	0.582	348
308	90.011	0.541	40	8.31	0.456	348
311	90.691	0.540	37	10.381	0.530	348
319	93.441	0.541	29	5.989	0.454	348
Mean	90.693	0.538	-	9.776	0.519	-
Standard deviation	10.2423	0.02958	-	2.10117	0.05204	-

Sensitivity analysis was conducted to assess the predictive strength of each input neuron, with results reported as normalized importance (Table 9). Vulnerability emerged as the most important predictor succeeded by response cost (68.54%), attitude (62.87%), response efficacy (61.35%), self-efficacy (46.67%), social influence (36.64%), and severity (35.16%).

**Table 9:** Importance of independent variables.

**Variables**	**Importance (Avg)**	**Importance (Norm)**	**Rank**
ATT	0.1529	62.87	3
PRE	0.1492	61.35	4
PS	0.0855	35.16	7
PSE	0.1135	46.67	5
PV	0.2432	100.00	1
RC	0.1667	68.54	2
SI	0.0891	36.64	6

PLS and ANN were compared (Table 10) to assess each independent variable's contribution to the dependent variable, as followed by Wang et al. (2022). Since severity, response efficacy, response cost, social influence, and attitude do not rank equally, it is possible that hidden qualities influence the functionalities of these variables. It may also be argued that a linear viewpoint of PLS alone could not adequately describe the relationships between the variables.

**Table 10:** Comparison between PLS and ANN.

**Relationships**	**|β| (PLS)**	**Rank (PLS)**	**NI (%)**	**Rank (ANN)**	**Remarks**
PS → AI	0.092	6	35.16	7	Not matched
PV → AI	0.314	1	100	1	Matched
PRE → AI	0.208	2	61.35	4	Not matched
RC → AI	0.171	3	68.54	2	Not matched
PSE → AI	0.105	5	46.67	5	Matched
SI → AI	0.055	7	36.64	6	Not matched
ATT → AI	0.133	4	62.87	3	Not matched

### 5.4 Classification algorithms

Machine Learning (ML) classifiers assess the variable importance and prediction robustness. These classifiers were not used to test the hypotheses but to evaluate the predictive accuracy and determine the relative importance of the variables in predicting the adoption intention. ML techniques capture non-linear relationships and offer higher prediction accuracy than PLS. This sequencing allows for a comprehensive analysis, where PLS provides insight into the relationships grounded in theory, and ML strengthens the robustness and predictive power of the model (AlHamad et al., 2021; Setrojoyo, 2024; Almarzouqi et al., 2022).

The classifiers' performance in predicting Adoption Intention is summarized in Table 11. Here, adoption intention is categorized into a 7-point Likert scale from strongly disagree to strongly agree. As per results, the Random Forest classifier demonstrated the highest overall effectiveness, predicting the AI with a correct classification rate (CCI) of 74.28%, and exhibiting balanced precision and recall metrics (0.74 for both), along with an F-measure of 0.72. The Random Forest model achieved the highest accuracy with a balanced precision–recall, demonstrating robust performance. These results suggest that the Random Forest model is highly effective at predicting IoT adoption intentions for home security in Dhaka. The high accuracy of 74.28% indicates that the model successfully classified the majority of respondents' adoption intentions, correctly identifying the respondents with a solid level of reliability. Additionally, the balanced precision and recall (both 0.74) demonstrate that the model is equally good at identifying both positive and negative instances of adoption, making it robust against class imbalances.

**Table 11:** Prediction of the AI by PS, PV, PSE, PRE, RC, ATT, and SI.

**Classifier**	**CCI (%)**	**Precision**	**Recall**	**F-measure**
Random forest	74.28	0.74	0.74	0.72
Decision tree	57.14	0.58	0.57	0.58
KNN	71.4	0.71	0.71	0.71
Gradient boosting classifier	72.85	0.73	0.73	0.71
SVM	68.57	0.69	0.69	0.68

## 6 Discussion

We have assessed the performance of PMT variables in our suggested model in comparison to some of the previous papers. Particularly in these publications, the PMT has been applied to information security. Furthermore, in line with our findings, these factors have served as a predictor of intention. Al-Emran et al. (2020), for instance, have expressed security issues regarding the usage of smart-watch for academic and learning objectives. Chen and Yeh (2017) have also discovered the factors that consumers consider while adopting smart meters to save energy. The findings on social influence were compared with Lee and Shin (2019) and Al-Momani et al. (2018), while results on attitude were contrasted with Hina et al. (2019) and Karahoca et al. (2018). According to Hina et al. (2019), PMT is used in the developing world to influence employees' security compliance behavior. Additionally, Karahoca et al. (2018) have sought to investigate important factors that influence people's propensity to adopt IoT.

Hypothesis H1 is supported, with perceived severity positively affecting adoption intention (β = 0.092, *p* < 0.05, *f*^2^ = 0.012). Al-Emran et al. (2020) disagree on the outcome, although Chen and Yeh (2017) concur. As we have predicted in our research, people consider burglary one of the greatest hazards, and losing valuables as a consequence of burglary may be quite upsetting for the victim. We may draw the conclusion that the prospective customers are aware of the scope of the potential burglary damages and, thus, are open to utilizing IoT devices. This characteristic, however, is found as the least substantial determinants of intention. This divergence suggests that in developing regions like Dhaka, where IoT for security is relatively new, users may prioritize the direct threat of burglary over abstract concerns of severity.

Hypothesis H2 is supported, with perceived vulnerability positively influencing adoption intention (β = 0.314, *p* < 0.001, *f*^2^ = 0.106) and representing the strongest predictor. This suggests participants are concerned about home security and motivated to adopt IoT-enabled devices due to perceived vulnerability. Al-Emran et al. (2020) obtained equal results from hypothesis test findings; however, they weren't comparable to Chen and Yeh's findings (Chen and Yeh, 2017). The result confirms the idea that consumers are more likely to adopt security-related technologies when they feel at risk. This supports the theoretical assertions of Protection Motivation Theory (PMT), which posits that threat perceptions are a key driver of protective behaviors (Lee and Shin, 2019). On the other side Dhaka faces significant crime rates, particularly burglary and theft, making residential security a primary concern. As a result, perceived vulnerability—the belief that one's home is at risk—becomes a strong predictor of IoT adoption intention, as individuals seek to protect their homes from prevalent security threats.

Hypothesis H3 is supported: response efficacy positively influences adoption intention (β = 0.208, *p* < 0.01, *f*^2^ = 0.046) and ranks as the fourth most important predictor (61.35%), consistent with Chen and Yeh (2017) and Al-Emran et al. (2020). So, people believe that adding IoT-enabled security measures is a successful strategy to prevent burglary. This aligns with the Protection Motivation Theory (PMT), which posits that individuals will engage in protective behaviors if they believe the response (i.e., IoT devices) will effectively mitigate the threat. However, our study's predictive accuracy shows that while response efficacy is important, it ranks lower than vulnerability, underscoring the necessity for robust communication of IoT's practical benefits.

Hypothesis H4, which posits a negative relationship between adoption intention and response cost, is supported (β = −0.171, *p* < 0.01, *f*^2^ = 0.041). The statistical significance of response cost is similar to those of Chen and Yeh (2017) and Al-Emran et al. (2020). Thus, it can be argued that potential users are discouraged from adopting security measures due to the perception that IoT technology is expensive. In other words, customers are less likely to freely adopt and use IoT technology since it requires extra time, effort, and financial resources. Additionally, the importance of this variable to adoption intention is ranked second. The relatively high response cost of IoT devices in Dhaka, where affordability and infrastructure can be barriers, may explain the negative relationship found here.

Hypothesis H5, which posits a positive effect of self-efficacy on intention, is not supported, as self-efficacy showed a negative effect (β = −0.105, *p*>0.05, *f*^2^ = 0.012). Nonetheless, its normalized importance remains 46.67%. Our hypothesis test findings regarding the relationship between self-efficacy and intention are disputed by Al-Emran et al. (2020) and Chen and Yeh (2017). Therefore, it does not seem that self-efficacy influences the decision to adopt an IoT device due to the following reasons. First, Hair et al. (2014) have identified a situation where security rules outperform technique reinforcement. Therefore, we can say that people are more knowledgeable about the effectiveness of IoT systems than about the actual process of utilization. Second, Brown et al. have identified that most potential users are unable to judge the appropriateness of the technology in their life. Similarly, all of the respondents in our survey are basically future users of the system who have not yet started using it. Therefore, it makes sense that they would doubt their own capacity to control the system. In contrast, research in more mature markets, such as those by Karahoca et al. (2018), demonstrates a stronger role for self-efficacy, likely because users have had more exposure to similar technologies and thus feel more confident in their use.

Hypothesis H6 is not supported: social influence has a positive but non-significant effect on adoption intention (β = 0.055, *p* > 0.05, *f*^2^ = 0.003) with a low normalized importance of 36.64%. This conclusion corresponds to Al-Momani et al. (2018) in terms of statistical significance, but not to Lee and Shin (2019). Therefore, one may contend that the adoption of IoT devices is not significantly influenced by social effects due to the following reasons. First, the respondents are unaware of how their acquaintances handle information security challenges, as claimed by Farooq et al. (2019). Second, social influence has less effect while technology is quite new, according to Venkatesh et al.. Similar to that, IoT and IoT-enabled security solutions are very new in Dhaka, and the population has not yet received enough education about them. This divergence might be attributed to the low level of familiarity with IoT security devices among the respondents in Dhaka. Since IoT is still in the nascent stage in Bangladesh, individuals may not yet see the behavior of others as a strong influence on their own adoption decisions. This finding suggests that social influence may become more important as IoT technology becomes more widespread and consumers become more educated about its benefits. In our case, IoT home security system is inherently a private, low-visibility device—typically installed inside the home and not publicly visible to peers or neighbors. As a result, social visibility and peer comparison mechanisms may be largely absent, which likely explains why SI did not emerge as significant in our model (Smith and Brown, 2022).

Hypothesis H7 is supported: attitude positively affects intention (β = 0.133, *p* < 0.01, *f*^2^ = 0.022) and ranks as the third most important predictor (62.87%). This moderate effect suggests that in developing countries like Bangladesh, the immediate need for security may outweigh general attitudes toward technology. This judgment is supported by Hina et al. (2019) and Karahoca et al. (2018). It can be said that participants consider this technology practical and helpful. In addition, the respondents express favorable attitudes toward implementing IoT technology, which can encourage implementing IoT technology. Moreover, this divergence highlights the cultural and market-specific factors that can shape how attitude impacts intention, suggesting that the relationship between attitude and adoption intention may vary depending on the maturity of the market, the technology in question, and the surrounding socio-economic context. The non-significant relationships for Social Influence and Self-Efficacy can be understood through the lens of Dhaka's cultural and socioeconomic context. In Dhaka, technology adoption decisions are often individual-driven and influenced by personal security concerns rather than social influence. Given the limited access to IoT devices and unequal distribution of wealth, many household heads prioritize perceived vulnerability over social norms. Social influence may not play a significant role, as IoT adoption remains more of a luxury for the affluent, and small social networks in Dhaka reduce broader societal influence. Similarly, the lack of significant self-efficacy can be attributed to socioeconomic factors such as limited exposure to technology and digital literacy. Lower-income households may lack both the resources and the confidence to adopt IoT security. Thus, personal risk perception and economic constraints are more influential than social factors in this context. In conclusion, while the study supports many of the findings from prior research, particularly regarding the importance of perceived vulnerability and response efficacy, it also highlights the unique context of Dhaka, where social influence and self-efficacy may not be as significant at this stage of IoT adoption. These findings suggest that marketing strategies in Dhaka should focus on addressing security concerns and lowering perceived costs, while social influence and self-efficacy may play a more prominent role as IoT adoption becomes more common and users gain familiarity with the technology.

This study combines the PLS and machine learning research approaches to evaluate the hypotheses and determine the importance of each element in predicting adoption intention. As per RO1, with *R*^2^ and *Q*^2^ being 34.9% and 17.9%, respectively, intention to adopt IoT is influenced by perceived severity, perceived vulnerability, response efficacy, response cost, and attitude. Moreover, in response to RO2, perceived vulnerability has the maximum contribution with 24.32% as followed by response cost, attitude, response efficacy, self-efficacy, social influence, and severity with 16.67%, 15.29%, 14.92%, 11.35%, 8.91%, and 8.55%, respectively, on an average. Finally, following the RO3, intention gained an accuracy of 74.28% with Random Forest classifier. The results indicate that Perceived Vulnerability and Response Efficacy are the most significant predictors of IoT adoption intention, highlighting the importance of security concerns in driving consumer behavior. This finding supports PMT's assertion that consumers are motivated to protect themselves from perceived threats, particularly in contexts such as home security, where personal safety and asset protection are paramount. The dominant role of Perceived Vulnerability suggests that, in Dhaka, individuals are primarily influenced by the perceived risk of security threats, such as burglary or theft, rather than by the technology itself.

In contrast, Social Influence and Self-Efficacy, which are key components of the ASE model, were found to have a minimal impact on adoption intention. This divergence from previous studies suggests that in developing markets like Dhaka, where IoT technology is still emerging, individuals may not yet be influenced by their social networks or confident in their ability to use the technology. The relatively low impact of Self-Efficacy could be explained by the novelty of the technology, where consumers may not have enough experience with IoT devices to form strong self-efficacy beliefs. Similarly, the lack of significant social influence may reflect the low awareness of IoT home security solutions, with individuals relying more on their personal perceptions of risk rather than social norms or peer behavior.

Furthermore, the Response Cost variable emerged as an important factor, indicating that economic considerations, such as the perceived financial and effort costs of IoT adoption, significantly affect decision-making. This highlights the need for affordable solutions and greater public awareness to lower perceived costs and enhance the adoption of IoT for home security. Overall, this study underscores the importance of risk perception in the adoption of security technologies, while also illustrating the evolving role of factors like attitude and self-efficacy in emerging markets. The findings suggest that future efforts to promote IoT adoption in Dhaka should focus on highlighting the security benefits of IoT devices and addressing perceived vulnerabilities, while also reducing the perceived costs of adoption. As the technology becomes more familiar, the role of self-efficacy and social influence may grow, offering avenues for future research to explore how these factors evolve over time.

Vitally, this study is one of the first ones to incorporate PMT and ASE in this particular research area. By increasing public awareness of home security, this study contributes to improving Dhaka's law and order while offering broader societal benefits. As the technological capabilities of the IT organization are rising in developing countries (Tello-Gamarra and Fitz-Oliveira, 2023), this study's findings are useful in defining the firms' investment and marketing strategies. As a consequence, future researchers might utilize the framework to evaluate IoT adoption in other developing nations.

## 7 Theoretical, methodological, and practical implications

By addressing several gaps in the literature, this paper makes a significant theoretical contribution. Specifically, by focusing on IoT, it extends the theoretical knowledge base within the field of information systems. IoT usage for home security has received little research interest. So, this study sheds insight into the variables influencing consumers' intention to adopt IoT-based security systems in their homes. Additionally, by examining user acceptance and creating a solid foundation for information system literature, this study helps to comprehension of IoT adoption for security goals. Second, to determine the elements that influence consumers' intentions to use IoT-based security systems, this study incorporated two significant theories, namely PMT and ASE. Indeed, the health domain is where both the PMT and the ASE models were born. Although PMT has previously been used in the field of information security, ASE's impact on the adoption of new technologies is quite modest. However, each of these models has been independently assessed in its respective field. Researchers have, however, missed combining these two models extensively. Most importantly, neither this suggested model nor Dhaka city has attempted to address security flaws in homes using IoT devices. The achieved variance and prediction accuracy of our suggested model is 34.9% and 17.9%, respectively. By combining PMT and ASE, this study provides a more holistic model for understanding IoT adoption. While PMT focuses on fear-driven motivation based on perceived threats (e.g., vulnerability to burglary), the ASE model complements this by showing how self-efficacy and attitudes toward technology mediate the adoption process. This dual framework enriches theoretical discussions by explaining not only the emotional triggers that drive adoption (e.g., fear of security threats) but also the cognitive factors (e.g., perceived ability and attitudes) that shape how individuals respond to those threats. The integration of these two models allows for a more nuanced understanding of how motivation and fear interact, which is crucial for explaining the decision to adopt IoT technology in security applications.

This study is among the first to apply a hybrid PLS-ANN approach to the PMT–ASE framework, offering a novel method to identify factors influencing IoT adoption for home security. ANN effectively captures nonlinear relationships with higher predictive accuracy than traditional methods like SEM (Leong et al., 2015). In addition, ANN does not need the fulfillment of any multivariate assumptions like normality, linearity, etc. (Leong et al., 2015; Zabukovšek et al., 2019). The ANN may thus be widely applied in information system research due to its powerful processing capacity and practical capability to anticipate adoption variables. From a methodological perspective, there are several significant advantages to using the hybrid PLS-ANN network model. First, this allows for supplementary verification of the SEM outcomes. On the other hand, this hybrid model offers a more accurate estimate of the relative effect of each predictor, allowing for the capture of both complicated linear and nonlinear interactions between antecedents and dependent variables (Zabukovšek et al., 2019). This reinforces the value of combining both analytical approaches, as they offer complementary insights, with PLS providing a clear understanding of direct relationships and ANN capturing intricate patterns that better reflects the dynamics of IoT adoption intention.

This research also makes some advances in practice. This study can increase people's awareness of home security, which can assist to strengthen Dhaka's overall law and order situation. Therefore, the entire society can gain if the prevalence of burglary and other domestic crimes is decreased via public awareness. Additionally, enhancing security awareness aligns with the Bangladesh Government's Digital Vision 2021 by promoting citizen safety (General Economics Division, 2012). It is anticipated that this research would be able to augment current policies with fresh insights and information. Conversely, consumer demand for IoT services is much lower than expected (Page et al., 2018). Additionally, the authors claim that consumer adoption is stalling and cannot proceed at the anticipated rate. The main factors influencing consumer IoT adoption, however, are prioritized in this study according to the impact they make. If these aspects are taken into account in investment and marketing strategies, consumer adoption will undoubtedly increase. This study can thus give businesses and entrepreneurs some helpful pointers. From a policy perspective, efforts should focus on reducing costs associated with IoT security devices and enhancing perceived response efficacy by educating consumers about the technology's effectiveness in mitigating security threats. For companies, marketing campaigns should emphasize trust in technology and highlight ease of use, addressing concerns about complexity and privacy to encourage adoption.

## 8 Limitations and future work

This research was undertaken in an IoT environment for security concerns. It is not apparent, therefore, if the results may be broadly applied to other kinds of applications. Additionally, it is possible that users in other nations won't have the same traits as the study's participants. In other countries, especially industrialized ones, the same factors might not be as crucial. On the other hand, only heads of families, who were adults, were allowed to submit data. However, the young (15–24 years) can be an interesting sample group and can be a crucial component in the adoption of any new technology, including the IoT. On the other hand, the intention of men and women might vary, and they could choose alternative factors for adoption (Albert et al., 2019). Consequently, a comparison between these groups could be able to add information to further knowledge study. This study employed snowball sampling combined with online survey administration, which may introduce selection bias by overrepresenting digitally connected and technology-aware household heads. As a result, households with limited internet access, lower digital literacy, or weaker social networks may be underrepresented. Consequently, the findings are not fully generalizable to the broader Dhaka population or to rural and less digitally engaged households. Future research should employ probability-based or mixed-mode sampling approaches to improve representativeness and external validity.

Other approaches to assessing the prediction accuracy can be tried during cross-validation. The first alternative can be 70% of data points for training and 30% for testing purposes whereas, in 2nd alternative, training, testing, and holdout can be 60%, 30%, and 10% respectively. On the other side, snowball sampling might introduce self-selection bias, social desirability bias and recall bias that might have influenced participants' responses and impact the generalizability as a whole. Finally, the focus of this study is limited to intention; therefore, actual behavior is also required to be investigated in future along with addressing controllability, risk, trust and privacy variable as moderator and mediator. Hence, future research may extend this framework by examining mediation or moderation mechanisms to further enrich theoretical understanding.

## 9 Conclusion

This study combines the PLS and machine learning research approaches to evaluate the hypotheses and determine the importance of each element in predicting adoption intention. As per RO1, with R2 and Q2 being 34.9% and 17.9%, respectively, intention to adopt IoT is influenced by perceived severity, perceived vulnerability, response efficacy, response cost, and attitude Moreover, in response to RO2, perceived vulnerability has the maximum contribution with 24.32% as followed by response cost, attitude, response efficacy, self-efficacy, social influence, and severity with 16.67%, 15.29%, 14.92%, 11.35%, 8.91%, and 8.55%, respectively, on an average. Finally, following the RO3, intention gained an accuracy of 74.28% with Random Forest classifier. The results indicate that Perceived Vulnerability and Response Efficacy are the most significant predictors of IoT adoption intention, highlighting the importance of security concerns in driving consumer behavior. This finding supports PMT's assertion that consumers are motivated to protect themselves from perceived threats, particularly in contexts such as home security, where personal safety and asset protection are paramount. The dominant role of Perceived Vulnerability suggests that, in Dhaka, individuals are primarily influenced by the perceived risk of security threats, such as burglary or theft, rather than by the technology itself.

In contrast, Social Influence and Self-Efficacy, which are key components of the ASE model, were found to have a minimal impact on adoption intention. This divergence from previous studies suggests that in developing markets like Dhaka, where IoT technology is still emerging, individuals may not yet be influenced by their social networks or confident in their ability to use the technology. The relatively low impact of Self-Efficacy could be explained by the novelty of the technology, where consumers may not have enough experience with IoT devices to form strong self-efficacy beliefs. Similarly, the lack of significant social influence may reflect the low awareness of IoT home security solutions, with individuals relying more on their personal perceptions of risk rather than social norms or peer behavior.

Furthermore, the Response Cost variable emerged as an important factor, indicating that economic considerations, such as the perceived financial and effort costs of IoT adoption, significantly affect decision-making. This highlights the need for affordable solutions and greater public awareness to lower perceived costs and enhance the adoption of IoT for home security. Overall, this study underscores the importance of risk perception in the adoption of security technologies, while also illustrating the evolving role of factors like attitude and self-efficacy in emerging markets. The findings suggest that future efforts to promote IoT adoption in Dhaka should focus on highlighting the security benefits of IoT devices and addressing perceived vulnerabilities, while also reducing the perceived costs of adoption. As the technology becomes more familiar, the role of self-efficacy and social influence may grow, offering avenues for future research to explore how these factors evolve over time.

Vitally, this study is one of the first ones to incorporate PMT and ASE in this particular research area. By increasing public awareness of home security, this study contributes to improving Dhaka's law and order while offering broader societal benefits. As the technological capabilities of the IT organization are rising in developing countries (Tello-Gamarra and Fitz-Oliveira, 2023), this study's findings are useful in defining the firms' investment and marketing strategies. As a consequence, future researchers might utilize the framework to evaluate IoT adoption in other developing nations.

## Data Availability

The raw data supporting the conclusions of this article will be made available by the authors, without undue reservation.
